# Complete genome sequence of *Bacillus thuringiensis* strain B-T-3 isolated from Qingdao, China, highly toxic against *Plutella xylostella* (Lepidoptera: Plutellidae)

**DOI:** 10.1128/mra.00174-26

**Published:** 2026-06-22

**Authors:** Kunhao Cai, Tianran Liu, Guiling Zheng, Qianlong Yu, Jie Li, Changyou Li

**Affiliations:** 1 Shandong Engineering Research Center for Environment-Friendly Agricultural Pest Management, College of Plant Health and Medicine, Qingdao Agricultural University 98431, Qingdao, Shandong, China; University of Manitoba, Winnipeg, Canada

**Keywords:** *Bacillus thuringiensis*, *Plutella xylostella*, whole-genome sequencing, insecticidal genes

## Abstract

*Bacillus thuringiensis* (Bt)-based biopesticides are crucial for the green control of agricultural pests. The Bt strain B-T-3 was isolated from Qingdao, China, and exhibits high toxicity against *Plutella xylostella* (Lepidoptera: Plutellidae). The complete genome sequence of B-T-3 and its plasmids and pesticidal protein gene contents was reported.

## ANNOUNCEMENT

*Bacillus thuringiensis* (Bt) is effectively used as a microbial biopesticide and widely utilized for controlling insect pests (1). Bt strain B-T-3 was isolated from the soil of a wasteland in Chengyang District, Qingdao, China, in 2009, by the sodium acetate selection method (2) and showed high insecticidal activity against *Plutella xylostella* (Lepidoptera: Plutellidae) larvae. Its virulence was evaluated via *in vivo* toxicity assays and showed a lethal concentration of 50% (LC_50_) of 7.92 × 10^6^ CFU/mL. The strain was deposited in the China Center for Type Culture Collection (CCTCC), Wuhan, China (collection number: CCTCC M 20252325).

For whole-genome sequencing (WGS), Bt strain B-T-3 was cultured in liquid Luria-Bertani (LB) medium at 28°C until the logarithmic growth phase (3, 4). The genomic DNA was extracted using a Bacteria Genomic DNA Kit (CoWin Biosciences, Inc., Taizhou, China) following the manufacturer’s instructions. The quality and concentration of the bacterial genomic DNA were evaluated via the Qubit 2.0 fluorometer and the Qubit dsDNA HS Assay Kit (Thermo Fisher Scientific Inc., MA, USA).

Whole-genome sequencing was performed using the Illumina NovaSeq 6000 platform (Illumina Inc., CA, USA) and the Nanopore PromethION platform (Oxford Nanopore Technologies [ONT], Oxford, UK) (5). For Illumina sequencing, libraries were prepared with the NEBNext Ultra DNA Library Prep Kit (New England Biolabs, MA, USA), assessed by the Qubit 4.0 Fluorometer (Thermo Fisher Scientific Inc.), and Qsep400 (BiOptic Inc., New Taipei City, Taiwan), and sequenced to produce 150 bp paired-end reads. Raw reads were processed with fastp v0.23.4 (6) using default parameters to remove adapters, trim low-quality bases, and discard short fragments, yielding 7,702,994 clean reads, totaling 1,146,948,726 bp, with a GC content of 35.04%. For Nanopore sequencing, size-selected DNA fragments (>1 kb) were processed with SQK-LSK110 and EXP-NBD196 kits. No DNA shearing was performed, and DNA repair, end preparation, barcode, and sequencing adapter ligation using Quick T4 ligase followed the manufacturer’s standard protocols. Sequencing was performed on an R9.4 flow cell (FLO-PRO002). Base calling used Guppy v.6.5.7 (high-accuracy model), and reads were filtered with NanoFilt v2.8.0 (7) with default settings, yielding 240,782 reads, totaling 1,971,135,637 bp, and an ONT read *N*_50_ of 14,072 bp.

Illumina and ONT reads were hybrid-assembled using Unicycler v0.4.9 (8) (parameters: -t 30 --linear_seqs 1) (https://github.com/rrwick/Unicycler/releases), in combination with SPAdes v4.0.0 (9) and Racon v1.3.3 (10). Overlaps were trimmed to yield circular replicons. Unicycler automatically rotated circular contigs to begin with *dnaA* or *repA* using tBLASTn. Genome completeness was evaluated with CheckM v1.1.6 (11), resulting in a completeness score of 98.92%. Plasmid sequences were independently assembled using the plasmid-specific tool Plassembler v1.8.0 (12), and all plasmid contigs were annotated using Prokka v1.14.6 (13). Additional validation of completeness, identity, and circularity of plasmids was performed via multiple sequence alignment with reference Bt strains HD-1 (ASM71753v1) (14), YBT-1520 (ASM74754v1), and HD73 (ASM33875v1) (15). Pesticidal protein gene content was mined via tBLASTn using pesticidal protein sequences retrieved from the Bacterial Pesticidal Protein Resource Center (https://www.bpprc-db.org) (16).

The genome comprises 1 circular chromosome (5,675,381 bp; 35.29% GC) and 11 circular plasmids (2,062–411,714 bp) (Table 1). The average short-read and long-read coverages were 202× and 347×, respectively. OrthoANIu analysis (https://www.ezbiocloud.net/tools/ani) (17) showed the strain had 99.80% ANI with *B. thuringiensis* subsp. *kurstaki* HD-1 (CP004870.1). Genome annotation results indicate that the genome of this strain contains a total of 5,784 predicted protein-coding genes. The pesticidal protein genes cover multiple families, including Cry, Vip, Vpb, and Mpp (Table 1).

**TABLE 1:** Sequence features and accession numbers of replicons from *Bacillus thuringiensis* strain B-T-3

Replicon	Accession number	Length (bp)	CDS	Pesticidal protein	GC content (%)
Chromosome	JBTFHN000000000	5,675,381	5,784	Spp1Aa	35.29
Plasmid 1	PX625142	411,714	353	Mpp46Ab, Vpb4Ca	32.61
Plasmid 2	PX651719	317,339	294	Cry1Aa, Cry1Ac, Cry1Ia, Cry2Aa, Cry2Ab, Vip3Aa	33.16
Plasmid 3	PX651720	82,531	103		30.98
Plasmid 4	PX651721	80,699	85		33.49
Plasmid 5	PX741347	69,355	66	Cry1Ab	35.15
Plasmid 6	PX741348	46,634	68		35.43
Plasmid 7	PX741349	14,889	26		31.11
Plasmid 8	PX741350	8,513	11		30.78
Plasmid 9	PX741351	8,279	11		29.77
Plasmid 10	PX741352	7,635	10		32.22
Plasmid 11	PX741353	2,062	2		34.86

## Data Availability

The raw reads have been deposited in NCBI and are available through the SRA database under accession number JBTFHN000000000, with the BioProject accession number PRJNA1402127.
